# Slower development of lower canopy beans produces better coffee

**DOI:** 10.1093/jxb/eraa151

**Published:** 2020-03-24

**Authors:** Bing Cheng, Heather E Smyth, Agnelo Furtado, Robert J Henry

**Affiliations:** 1 Queensland Alliance for Agriculture and Food Innovation, The University of Queensland, St Lucia, QLD, Australia; 2 RWTH Aachen University, Germany

**Keywords:** Canopy, coffee, development stages, gene expression, metabolism pathways, sucrose, transcriptome

## Abstract

The production of high-quality coffee is being challenged by changing climates in coffee-growing regions. The coffee beans from the upper and lower canopy at different development stages of the same plants were analyzed to investigate the impact of the microenvironment on gene expression and coffee quality. Compared with coffee beans from the upper canopy, lower canopy beans displayed more intense aroma with higher caffeine, trigonelline, and sucrose contents, associated with greater gene expression in the representative metabolic pathways. Global gene expression indicated a longer ripening in the lower canopy, resulting from higher expression of genes relating to growth inhibition and suppression of chlorophyll degradation during early bean ripening. Selection of genotypes or environments that enhance expression of the genes slowing bean development may produce higher quality coffee beans, allowing coffee production in a broader range of available future environments.

## Teaser

The lower canopy was found to produce better quality coffee with slower maturity. Candidate genes associated with slow bean maturity and improved coffee quality were characterized.

## Introduction

Climate change has resulted in various environmental changes, such as temperature, drought, and increased CO_2_, that affect plant growth and the final quality of agricultural and food products (Verhage *et al.*, 2017; Rahn *et al.*, 2018). With a high demand for climate-resilient products, genetic improvement is considered as a key approach in adapting plants to environmental change (Henry, 2019). Recent concerns about the extinction of wild species due to climate change further emphasize the importance of understanding the genetic and environmental basis of product quality, as the wild species are essential genetic resources for sustainable production and improvement of quality (Davis *et al.*, 2019).

Coffee is one of the most important cash crops in the third world and has substantial economic value (Fridell, 2014). It is often used as a typical example in climate change studies due to the dramatic influences that already and will further threaten coffee production (DaMatta et al., 2016, 2019; Ramalho *et al.*, 2018; Semedo *et al.*, 2018; Worku *et al.*, 2018). The current area of coffee plantings will decrease by ~50% by 2050 (Moat *et al.*, 2017). However, recent research has clearly demonstrated that coffee plants can endure temperatures much higher than traditionally estimated and that the increase in atmospheric CO_2_ (which is expected to increase in parallel and promote the temperature rise) is a key factor in coffee plant acclimation to high temperature, strengthening coffee metabolism in key pathways and antioxidant protection, and modifying gene transcription and mineral balance (DaMatta et al., 2016, 2019; Martins *et al.*, 2016; Rodrigues *et al.*, 2016; Verhage *et al.*, 2017; Rahn *et al.*, 2018). This makes coffee an exceptional case for use in exploring the mechanism of plant adaptation to the changing environment.

Environmental influences on coffee quality have been explored in depth in studies of the variation of chemical composition and the candidate genes from the related metabolic pathways (Muschler, 2001; Joët, 2009, 2010*a*, *b*; Odeny *et al.*, 2014; Cheng *et al.*, 2016). However, knowledge of the developmental and environmental regulation of coffee quality and related metabolic pathways is limited. Moreover, patterns of co-expression may also influence the final composition. For example, other than just being an essential flavour precursor, sucrose is also the main contributor of the carbon backbone of many key components in the cell, such as cell wall polysaccharides, proteins, and lipids (Ruan, 2014; Cheng and Henry, 2019).

Transcriptome analysis provides a tool that can be used to explore the relationships between genes, their expression, and the composition and quality attributes of coffee beans. Transcriptome analysis is a straightforward approach and it is widely applied to capture gene expression (Denoeud *et al.*, 2014; Dal Santo *et al.*, 2018; Henry *et al.*, 2018). Replicated RNA sequencing (RNA-Seq) provides a quantitative estimation of gene expression for specific transcripts and avoids the need for real-time PCR (RT-PCR) confirmation of microarray results (Liu *et al.*, 2014; Lamarre *et al.*, 2018; Ramírez-González *et al.*, 2018; Zhang *et al.*, 2018). RNA-Seq is especially useful in polyploid genomes such as Arabica coffee, as microarrays and RT-PCR do not always distinguish many different closely related transcripts that are found in polyploids (Hoang *et al.*, 2017; Ramírez-González *et al.*, 2018). Using the same sample set, we had generated the first long-read coffee bean transcriptome that provided a platform for transcriptome analysis in this study Cheng *et al.* (2017).

A gap remains in our understanding of the path from flavour profile to chemical composition, and especially to genetic fingerprint. As a perceived key quality, flavour is critical to consumer acceptance of food products (Brückner and Wyllie, 2008). The flavour profile of processed food products, derived from flavour precursors, is inherently associated with the genetics of the plant (Cheng *et al.*, 2016). We adopted a consumer-focused approach to understand the genetic and compositional basis of product quality. Other than sucrose, many metabolites determine coffee flavour, such as caffeine and trigonelline. Caffeine provides perceived strength and body to the coffee beverage as well as bitterness (Janzen, 2010), and is associated with good quality (Farah *et al.*, 2006). Trigonelline and sucrose are important flavour precursors, with the majority of them being degraded during roasting to form flavour components (including pyridine, pyrroles, aldehydes, and carboxylic acids) (Clarke and Macrae, 1985; Clifford, 1985; Dart and Nursten, 1985; De Maria *et al.*, 1996; Janzen, 2010). Trigonelline contributes to the overall aroma perception and bitterness, and was found to be strongly correlated with high coffee quality (Farah *et al.*, 2006).

Growth environment is a combination of many factors, including light, temperature, soil, and water availability (Wintgens, 2004). To reduce some of this complexity, we studied coffee beans from two levels of the same tree to compare the impact of the microenvironment at different canopy levels on coffee quality (see Supplementary Fig. S1 at *JXB* online). Analysis of beans from different canopy positions in the same plant allows the influence of this environmental factor to be considered in the absence of genetic effects. Coffee beans from different ripening stages, immature (green pericarp), intermediate (yellow), and mature (red) stages, were analysed to define the transcriptome and canopy position (above and below 1.7 m) influences on coffee quality. Results from this study should provide new strategies to produce high-quality coffee.

## Materials and methods

### Coffee bean processing

Four biological replicates of mature *Coffee arabica* L. K7 fruits (~5 kg) were harvested from the upper (>1.7 m) and lower (<1.7 m) tree canopy (3–4 m high) (Supplementary Fig. S1). Sample collection was conducted between 10.00 h and 14.00 h in October 2015 at the coffee farm of Green Cauldron Coffee, Federal, New South Wales, Australia. A dry-processing method was applied in this study. Harvested coffee cherries were first de-pulped with a coffee huller (CAPE, Australia) and sun-dried. When samples reached a moisture content of ~12%, a de-husking process was performed to obtain raw coffee beans. Raw beans were then split into two groups: one set was used for physicochemical analysis, including bean size, weight, caffeine, trigonelline, and sucrose analysis. The other set was roasted at 193 °C for 10 min using a Probatino coffee roaster (Probat, Germany) and assessed by sensory evaluation.

### Phenotypic analysis

#### Sensory evaluation

Sensory preparation, training, and evaluation were guided by Australian Standard AS 2542 1.1 and standard sensory description (Smyth *et al.*, 2012).

##### Sample preparation and presentation.

Roasted samples of coffee beans from the upper and lower canopy were prepared for sensory assessment such that panellists could compare treatments as both whole beans and freshly ground bean samples. The whole bean (unground) sample was prepared with one bean from each biological replicate and four beans in total for the upper or lower canopy whole bean samples. For ground coffee sample assessment, coffee beans were ground and a portion (0.5 g) was placed in a clear plastic 60 ml serving cup sealed with a lid.

##### Preliminary screening.

Prior to the formal evaluation, a preliminary sensory screening was performed whereby samples of ground coffee and coffee beans were presented to a panel of 12 trained tasters. The panellists had been previously recruited externally after pre-screening for sensory acuity and training in sensory methods, and were experienced in participating in descriptive analysis studies of food and beverage products. The trained sensory panellists were asked to describe the aroma and appearance of samples and indicate any qualitative or quantitative sample differences, followed by a discussion session to reach a consensus on the key sample differences. A sensory lexicon for coffee was provided to the panel (Supplementary Table S1).

##### Difference testing.

Formal evaluation of samples was conducted using a paired comparison difference test (ISO, 2002; Prescott *et al.*, 2005) involving a total of 86 participants who were staff and students from the Health and Food Sciences Precinct, Coopers Plains, Brisbane, Australia. A number of sessions were conducted throughout two subsequent days with up to 12 participants attending each session. As per the paired comparison test, samples were presented to panellists according to a balanced design in pairs, and participants were asked to evaluate the aroma and identify the sample with a stronger (more intense) aroma. The paired comparison is a forced-choice technique. Data were obtained and processed with Compusense^®^ five software (5.0.49, Compusense Inc., Guelph, Ontario, Canada) based on a two-sided directional paired comparison (Tikapunya *et al.*, 2018).

Sensory evaluation was conducted in the purpose-built sensory laboratory and focus group room of the Health and Food Sciences Precinct, Coopers Plains, Brisbane, Australia. The 12 individual booths were temperature and light controlled and equipped with computers for data collection. The focus group room, used for preliminary screening, was a boardroom-style set up with a round table, chairs, and a smartboard for collecting information from discussions.

#### Bean size and weight measurement

For each of the four biological replicates, the length, width, and thickness of coffee beans were measured by a Vernier caliper with 10 raw beans, and bean weight was determined by measuring 100 raw coffee beans using an analytical balance.

#### Analysis of caffeine and trigonelline

Caffeine and trigonelline extraction were performed as previously described with some modifications (Casal *et al.*, 1998). Green coffee powder (2 g) was extracted after boiling in MiliQ water (20 ml) while mixing for 2.5 min. The extraction process was repeated twice, and the total volume adjusted to 100 ml. Then the solution was cooled in an ice bath and centrifuged (1500 *g*, 4 °C, 5 min) before filtering with a 0.45 μm syringe filter (Thermo Fisher Scientific, Australia) and transferring to 2 ml HPLC vials (Thermo Fisher Scientific, Australia). Extractions were performed in triplicate. Samples were stored at –80 °C in HPLC vials until analysis could take place.

The analysis of caffeine and trigonelline was performed using HPLC as described previously (Casal *et al.*, 2000). Calibration was performed with caffeine and trigonelline standards between 0.5 µg ml^–1^ and 500 µg ml^–1^. Reagents caffeine and trigonelline hydrochloride (analytical standards), and methanol and KH_2_PO_4_ (analytical grade) were purchased from Sigma-Aldrich (USA). Extracts were analysed with a Waters Associates (USA) HPLC, filtered with a Spherisorb S5 ODS2 (0.46×25.0 cm) column and a Bondapak C18 (10 µm) guard column. Gradient A was a phosphate buffer (KH_2_PO_4_) 0.1 M (pH 4.0), while B was methanol at 0 min (7%), 4 min (9%), 6 min (25%), 13 min (29%), 21 min (50%), and 35–40 min (7%). The injection volume was 20 µl. The UV detection was set at 265 nm.

#### Analysis of sucrose

The method used for the extraction of sucrose was modified from a previous study (Abid *et al.*, 2014). Green coffee (ground) samples (0.1 g) were mixed with 4 ml of MiliQ water. After sonication (20 min), extracts were centrifuged and filtered prior to HPLC analysis. Extraction was performed in duplicate.

The method used for HPLC analysis of sucrose followed a procedure validated previously (Ma *et al.*, 2014). Calibration was performed with a sucrose standard (Sigma Aldrich) at concentrations between 100 mg ml^–1^ and 500 mg ml^–1^. An Agilent HPLC 1110 series (ELSD detection) was used fitted with an Alltech Prevail Carbohydrate ES (5 μm, 250 mm×4.6 mm) column. The mobile phase used was 25% water and 75% acetonitrile isocratic. The injection volume was 10 µl.

### Statistical analysis for phenotypic analysis

The significant analysis on the above phenotypic analysis was carried out with the single-factor ANOVA analysis tool (alpha=0.05) built in Microsoft Excel 2016, except for sensory evaluation analysis.

### Transcriptome analysis

#### RNA sample and cDNA library preparation

Three biological replicates of coffee cherries, *C. arabica* L. K7, from three developmental stages, green [green pericarp, ~180 days after flowering (DAF)], yellow (yellow pericarp, ~210 DAF), and red (red pericarp, ~240 DAF) stages (cherries only from upper or lower canopy) using the same sample, RNA extraction, and cDNA preparation as reported previously (Cheng *et al.*, 2017) (Supplementary Fig. S1). Five coffee fruits were randomly picked from each stage and canopy position. One biological replicate contains the collection from five trees in total. Altogether, 18 samples of 450 coffee fruits (900 beans) were harvested for transcriptome analysis. Sample harvest was performed between 10.00 h and 14.00 h on 11 August 2015 from the same coffee farm as coffee bean processing, at Green Cauldron Coffee, Federal, New South Wales, Australia. When coffee fruits were selected, the pericarp was removed using a scalpel and discarded within 20 s, and then beans inside were immediately frozen in liquid nitrogen. Frozen beans were transported to the laboratory on dry ice and stored in –80 °C until further use.

Coffee beans were pulverized with a TissueLyser II (Qiagen, Germany) in an environment with liquid nitrogen. Total RNA isolation was carried out according to the protocol established previously (Furtado, 2014). The integrity of total RNA was accessed with an Agilent RNA 6000 nano kit and chips through a Bioanalyzer 2100 (Agilent Technologies, CA, USA). Thereafter, a standard 18× Truseq total RNA library was prepared with an additional Ribo-Zero kit. Samples were subsequently sequenced on an Illumina HiSeq4000 platform (2×150 bp paired-end reads). One of the replicates from the lower canopy at the yellow stage was contaminated, so it was not further analysed thereafter. Data from the upper canopy were adopted from our previous study (Cheng *et al.*, 2018) on the identification of key chemical accumulation in different developmental stages.

#### Read mining and RNA-Seq analysis

Raw reads were mainly processed with CLC Genomic Workbench 10.0.1 (CLC, QIAGEN, CLC Bio, Denmark) as follows. (i) Raw read quality was accessed and then adapters and indexes were trimmed. (ii) A further trimming with quality (0.01) and length (≥40 bp) was conducted and read quality was accessed. (iii) Trimmed reads (both singletons and paired reads) were processed through RNA-Seq analysis. The coffee bean long-read sequencing transcriptome was used, based upon the same sample collection and total RNA preparations (Cheng *et al.*, 2017). Transcripts per kilobase million (TPM) was selected for use as expression values.

#### Statistical analysis

For transcriptome analysis, only genes with TPM >1 were considered as valid for expression analysis. Candidate genes studied in the metabolism pathways of caffeine, sucrose, and trigonelline were based on our previous studies (Cheng et al., 2016, 2017). Statistical analysis of candidate genes was according to the ANOVA single-factor test (alpha=0.05) tool built in Microsoft Excel. For clarity, abbreviations of different sample names will be used hereafter when applicable. UG, UY, and UR stand for upper canopy coffee beans at the green, yellow, and red stages; LG, LY, and LR are lower canopy coffee beans at the green, yellow, and red stages.

Differential gene expression was analysed with the tool built for RNA-Seq analysis using CLC. Two comparisons were conducted in this study. (i) To compare genes expressed at different canopy positions, three subcomparisons were made: UG versus LG, UY versus LY, and UR versus LR. Differentially expressed genes (DEGs) from these comparisons were annotated with the LRS coffee bean transcriptome reference, and the annotation quality was filtered with a bit score of >300. The expression values of DEGs were filtered with a false discovery rate (FDR) *P*-value <0.01 and maximum group mean (maximum mean of gene expression in any replicate of the three developmental stages) >10. 2. Variations in different developmental stages of the upper and lower canopy positions were assessed separately, including UY versus UG, UR versus UY, LY versus LG, and LR versus LY. DEGs from these comparisons were processed with Mercator for the classification of photosynthesis-, hormone-, and stress-associated genes (Schwacke *et al.*, 2019). Classified DEGs were prepared for the construction of the co-expression network with caffeine, trigonelline, and sucrose genes. Co-expression network analysis was based on DEGs from both comparisons and processed through (WGCNA) built in MEV (weight >0.85) and visualized by Cytoscape 3.5.1 (Shannon *et al.*, 2003; Howe *et al.*, 2010; Li *et al.*, 2018).

## Results

### Coffee aroma

A sensory evaluation provides a straightforward approach to the determination of the quality of coffee. The preliminary sensory screening revealed more intense aroma in the beans from the lower canopy (Table 1). Whole beans from the upper canopy had low aroma intensity, while those from the lower canopy were described by the panel as having a medium aroma intensity. The aroma of beans from the upper canopy (unground beans) was uniquely described as smelling of milk, sweet, caramel, fresh grassy, woody, and old perfume, while the beans from the lower canopy were described as strong, roasted, fruity (citrus, raspberry, lime), beef jerky, and chrysanthemum. Not surprisingly, a much stronger aroma was produced by ground coffee compared with the whole unground bean samples. The aroma of the ground beans from the upper canopy was described as pipe tobacco, herby, woody, earthy, mushroom, and dry dusty. The aroma of the ground beans from the lower canopy was distinguished as having a higher intensity and a deeper aroma, described as dark chocolate, orange, and citrus zest. Thereafter, a difference test (paired comparison) was conducted which indeed demonstrated that beans from the lower canopy had a significantly more intense aroma compared with those from the upper canopy (*P*-values of <1% and <5% for ground and whole beans, respectively).

**Table 1.** Summary of the qualitative descriptions given by the trained panel during a screening of whole and ground coffee beans from the upper and lower canopy (*n*=12)

Whole bean	Ground bean		
Upper canopy	Lower canopy	Upper canopy	Lower canopy
**Low aroma intensity**	**Medium intensity**	**Medium odour intensity**	**Higher intensity**
Milk, vanilla, sweet candy, brown sugar, caramel, treacle, golden syrup, malt, burnt, Brazil nut, berry, smoky, fresh, grassy, pepper, spice, milk-chocolate, old perfume/cologne, woody, flowery	Hazelnut, roasted, nut, smoky, tobacco, strong, chico babies, cinnamon, spice, chocolate, lime, pepper, malt, vanilla, citrus, raspberry, beef jerky, Chinese medicine, mulberry/ chrysanthemum, flowery	Herby, caramel, roasted nuts, intense, onion/pepper, chocolate, burnt, toast, woody, pipe tobacco, blackcurrant, molasses, jammy, mushroom, earthy, dry dusty, Szechuan pepper, tree sap, floral, smoky	Deeper, dark chocolate, smoky, charred, toast, nutmeg, nutty, cedar, burnt, citrus zest, spicy, BBQ spice, mixed spice, orange, treacle

### Bean size and chemical composition

**Fig. 1.**
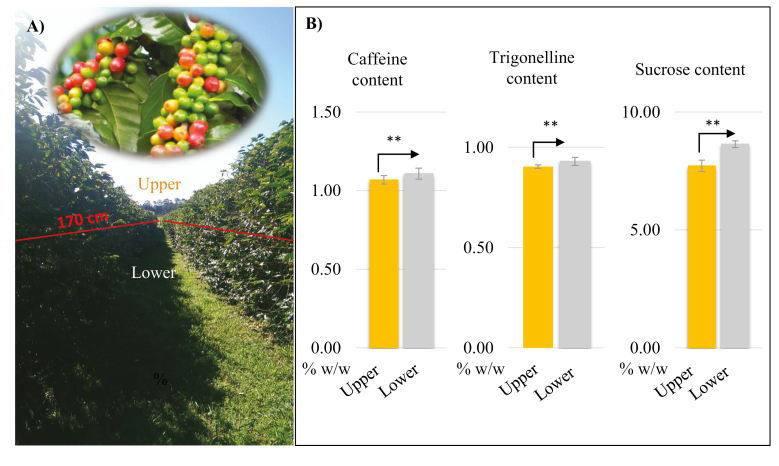
Chemical components of coffee beans (caffeine, trigonelline, and sucrose) as influenced by canopy position. (A) Coffee beans (*Coffea arabica* L. K7) were collected from the lower and upper canopy (above and below 170 cm). (B) Histogram of the caffeine, trigonelline, and sucrose content in coffee beans from the upper and lower canopy (as a percentage of dry matter) (*n*=4, mean ±SD). ** indicates *P*<1%.

There was no significant difference (*P*-value >5%) in bean size and weight between beans from the upper and lower canopy (Table 2). Beans from the lower canopy were significantly higher in caffeine (1.07±0.07 versus 1.11±0.04 g 100 g DW^–1^), trigonelline (0.90±0.01 versus 0.93±0.02 g 100 g DW^–1^), and sucrose (7.7±0.2 versus 8.6±0.1 g 100 g DW^–1^) content (*P*<1%) compared with those from the upper canopy (Fig. 1B).

**Table 2.** Bean weight and size in coffee beans from the upper and lower canopy have no significant differences (*n*=4, mean ±SD, *P*-value >5%)

Coffee bean samples	Weight g 100^–1^ beans	Bean size		
		Length (mm)	Width (mm)	Thickness (mm)
Upper	18.6±1.0	9.6±0.6	7.5±0.4	4.0±0.2
Lower	19.2±0.3	9.7±0.6	7.2±0.4	4.1±0.3
*P*-value	0.1	0.4	0.2	0.2

### Gene expression and bean composition

**Fig. 2.**
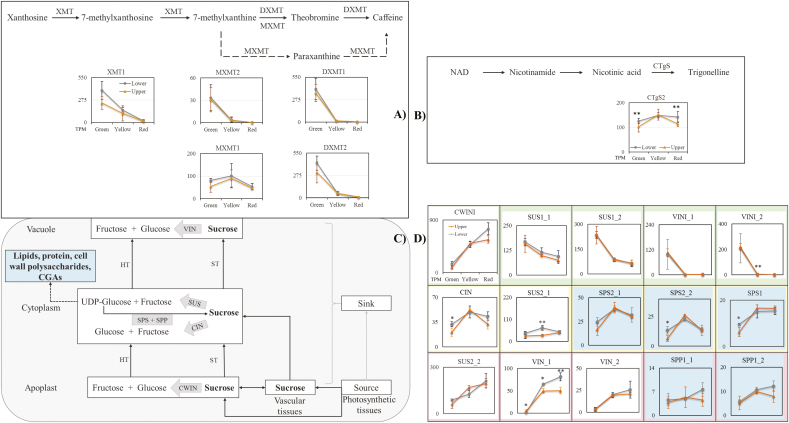
Difference in gene expression of different metabolism pathways. (A) Caffeine biosynthesis pathway. (B) Trigonelline biosynthesis pathway. (C) Sucrose metabolism pathway. (D) Gene expression in the sucrose metabolism pathway. XMT, 7-methylxanthosine synthase; MXMT, 7-methylxanthine methyltransferase; DXMT, 3,7-dimethylxanthine *N*-methyltransferase; CTgS2, trigonelline synthase 2; SUS, sucrose synthase; VIN, vacuolar invertase; CIN, neutral invertase; CWIN, cell wall invertase 4; VINI, vacuolar invertase inhibitor; CWINVI1, cell wall invertase inhibitor; SPS, sucrose phosphate synthase; SPP, sucrose phosphate phosphatase. Note: TPM±SD was used in each time point; *, **, significant difference (*P*<5%, *P*<1%) between the upper and lower canopy at individual ripening stages; _1 and _2, different transcript isoforms identified.

To explore the reason for the increase of these components in the beans from the lower canopy, the expression of genes associated with caffeine, trigonelline, and sucrose metabolism pathways was analysed. Expression of genes in the caffeine biosynthetic pathway was highest at the green bean stage (Fig. 2A), with the exception of *MXMT1* (encoding 7-methylxanthine methyltransferase 1 which regulates the alternative caffeine biosynthetic pathway) that peaked at the yellow stage. This indicates that caffeine was mainly accumulated at early stages of bean maturation. However, no significant difference was observed in the gene expression associated with caffeine biosynthesis for the two canopy positions. Trigonelline synthase was expressed more at the green and red stages in coffee beans in the lower canopy compared with those from the upper canopy (Fig. 2B, *P*<5%). This suggests that trigonelline accumulation started earlier and finished later.

Sucrose is transferred from the photosynthetic source tissues to the sink tissues through the apoplast (through the cell) and symplast pathways (through the cytoplasm). In sink tissues, sucrose is degraded by various tissue-specific invertases to hexoses (Fig. 2C). These invertases include cell wall invertase (CWIN, apoplast), cytosolic invertase (CIN), and vacuolar invertase (VIN). In the cytoplasm specifically, sucrose is degraded by sucrose synthase (SUS) to UDP-glucose, which can be used for cell wall polysaccharide synthesis or sucrose resynthesis by sucrose phosphate synthase (SPS) and sucrose phosphate phosphatase (SPP). The expression of genes in the sucrose metabolism pathways suggested flow of gene expression from the cell wall (mainly at the green stage) to the cytoplasm (yellow and red stages) and then to the vacuole (red stage). *CWINI* (maximum 748±102 in LR) and *SUS1* (maximum 235±53 in UG) were both expressed at a very high level, suggesting their important role in sucrose metabolism and cell development. Sucrose degradation by CIN and SUS2 and resynthesis by SPS and SPP were greatest at the yellow and red stages. Sucrose degradation by VIN was highest in the vacuole at the red stage. Significantly, the elevated sucrose level in coffee beans from the lower canopy probably resulted from increased expression of *SPS1*, *SPS2_2*, *SUS2_1*, *CIN*, *VIN_1*, and *VINI_2* (Figs 1, 2D). Higher *CIN* and *SUS2_1* expression in the lower canopy at the green and yellow stage suggested that more sucrose was loaded from source tissues combined with a more rapid degradation to produce more downstream components in the cell wall and cytoplasm. Consistent expression of *SPS1* and *SPS2_2* in the lower canopy at the green stage (*P*<5%) suggested more sucrose being formed using the degraded components from CIN and SUS2_1. Large differences between the upper and the lower canopy, decreases (UG versus LG) and increases (UY versus LY and UR versus LR), were identified in *VIN_1* expression in the lower canopy, This indicated that more sucrose was loaded into the sink tissue of UG at early bean development and then more was transported to LY and LR, where sucrose was degraded for downstream biosynthesis of components in the vacuole. In contrast, significantly higher expression was seen in *VINI_2* for beans from the lower canopy at the yellow stage, negatively associated with the expression trend of *VIN_1*.

**Fig. 3.**
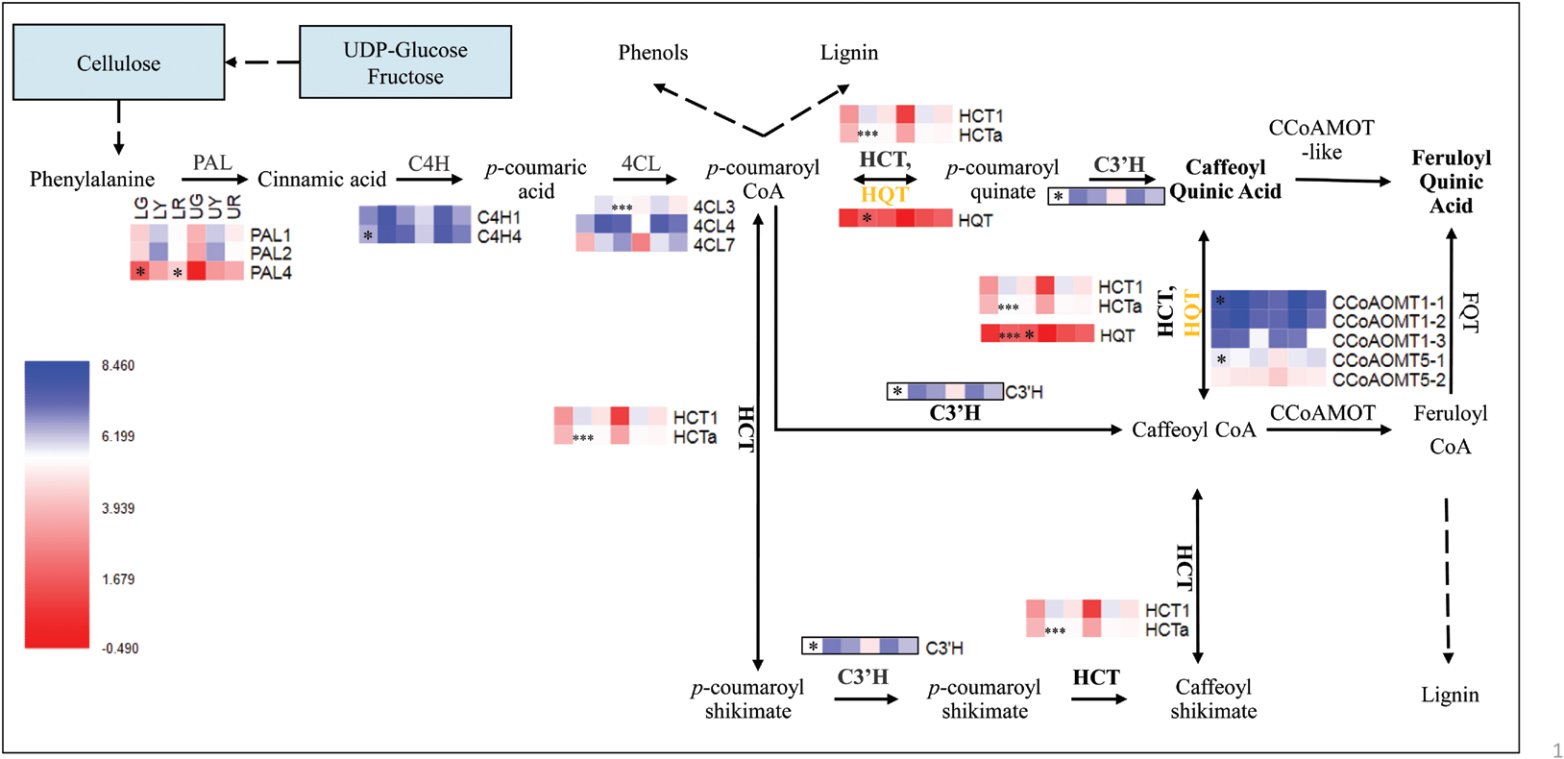
Gene expression in the major chlorogenic acid biosynthetic pathways, adapted from Cheng *et al.* (2016). LG, LY, and LR indicate coffee beans at the green, yellow, and red stages from the lower canopy; UG, UY, and UR indicate coffee beans at the green, yellow, and red stages from the upper canopy; PAL, phenylalanine ammonia lyase; C4H, trans-cinnamate 4-hydroxylase; 4CL, 4-coumarate: CoA ligase; HCT, hydroxycinnamoyl-CoA:shikimate/quinate hydroxycinnamoyl transferase; HQT, hydroxycinnamoyl-CoA quinate hydroxycinnamoyl; C3'H, *p*-coumaroyl CoA 3-hydroxylase; CCoAOMT, caffeoyl-CoA 3-*O*-methyltransferase. ***P-value <0.1%, *P-value <5%.

What was sucrose degradation used for? From the genes regulating key components in the bean, we can have an overview of knowledge. These genes include those encoding enzymes in the major chlorogenic acid (CGAs; Fig. 3) and cell wall polysaccharide metabolism pathways (Supplementary Fig. S2), as well as key genes regulating lipids and protein formation (Supplementary Fig. S3). At the green stage, the degradation of sucrose mainly leads to the biosynthesis of major bean components. This includes galactomannan (main cell wall polysaccharides), triacylglycerol (major lipids), and 11S globulin (main protein), as suggested by the expression of *mannose 1 phosphate guanylyltransferase 1*, *mannan synthase 1*, *galactomannan galactosyltransferase 1–2*, *alpha-galactosidase 1–2*, *oleosin proteins 1–5*, and *11S 1–2*. From the yellow to the red stage, cell wall polysaccharides and proteins were modified, and new types were formed according to the expression of *cellulose synthase A1–3* and *galacturonosyltransferase*. Biosynthesis of CGAs is another route for utilizing sucrose degradation compounds. According to gene expression in the CGA metabolism pathway, in LG, the increase of gene expression was in *PAL4*, *C4H4*, *C3'H*, *CCoAOMT1*, and *CCoAOMT5*. At the yellow stage, an improvement was shown in *4CL3*, *HCTa*, and *HQT*. At the red stage, only two genes had a greater expression, *PAL4* and *HQT*.

### Transcriptome analysis of influences of canopy position

**Fig. 4.**
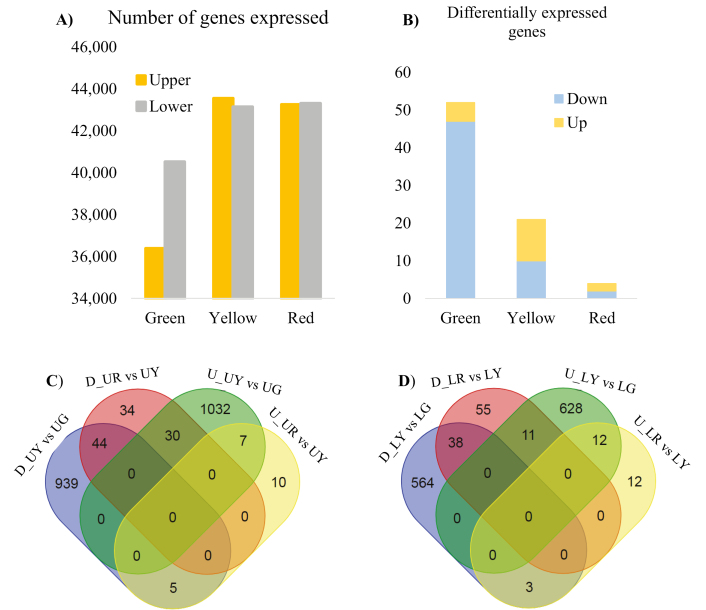
Transcriptome analysis of developing coffee beans from the upper and lower canopy. (A) Genes expressed in coffee bean ripening from the lower and upper canopy. (B) Differentially expressed genes (DEGs) in the upper and lower canopy comparison through different ripening stages (up-/down-regulated in coffee beans from the lower canopy). (C) The number of upper canopy DEGs that were up-/down-regulated in the comparison of the yellow versus green stage (UY versus UG) and red versus yellow stage (UR versus UY). (D) The number of lower canopy DEGs that were up-/down-regulated in the comparison of yellow versus green stage (LY versus LG) and red versus yellow stage (LR versus LY). Note: the upper canopy data were extracted from ENA with accession number: PRJEB24137.

One aim of the transcriptome analysis was to identify genes influenced by canopy position that were associated with phenotypic variation. The number of genes expressed in beans from the lower canopy showed a steady increase from the green to red stages (Fig. 4A), while the number of genes expressed in beans from the upper canopy peaked at the yellow stage. A higher number of genes were expressed in the beans of the lower canopy at both the green and red stages. More DEGs were observed in the green stage comparison between the two canopy positions (Fig. 4B), with the majority of the DEGs being down-regulated in the lower canopy. Greater changes were observed in the upper canopy from the green to the yellow stage. This is consistent with the number of DEGs from different stages. More up- and down-regulated DEGs were found in the comparison of yellow versus red stage in the lower compared with the upper canopy (Fig. 4C, D). According to the principal component analysis (PCA) of overall gene expression, fewer changes were shown in the yellow and red stages (Supplementary Fig. S4). Moreover, compared with gene expression in the upper canopy, lower canopy samples varied less.

### Quality-associated genes

**Fig. 5.**
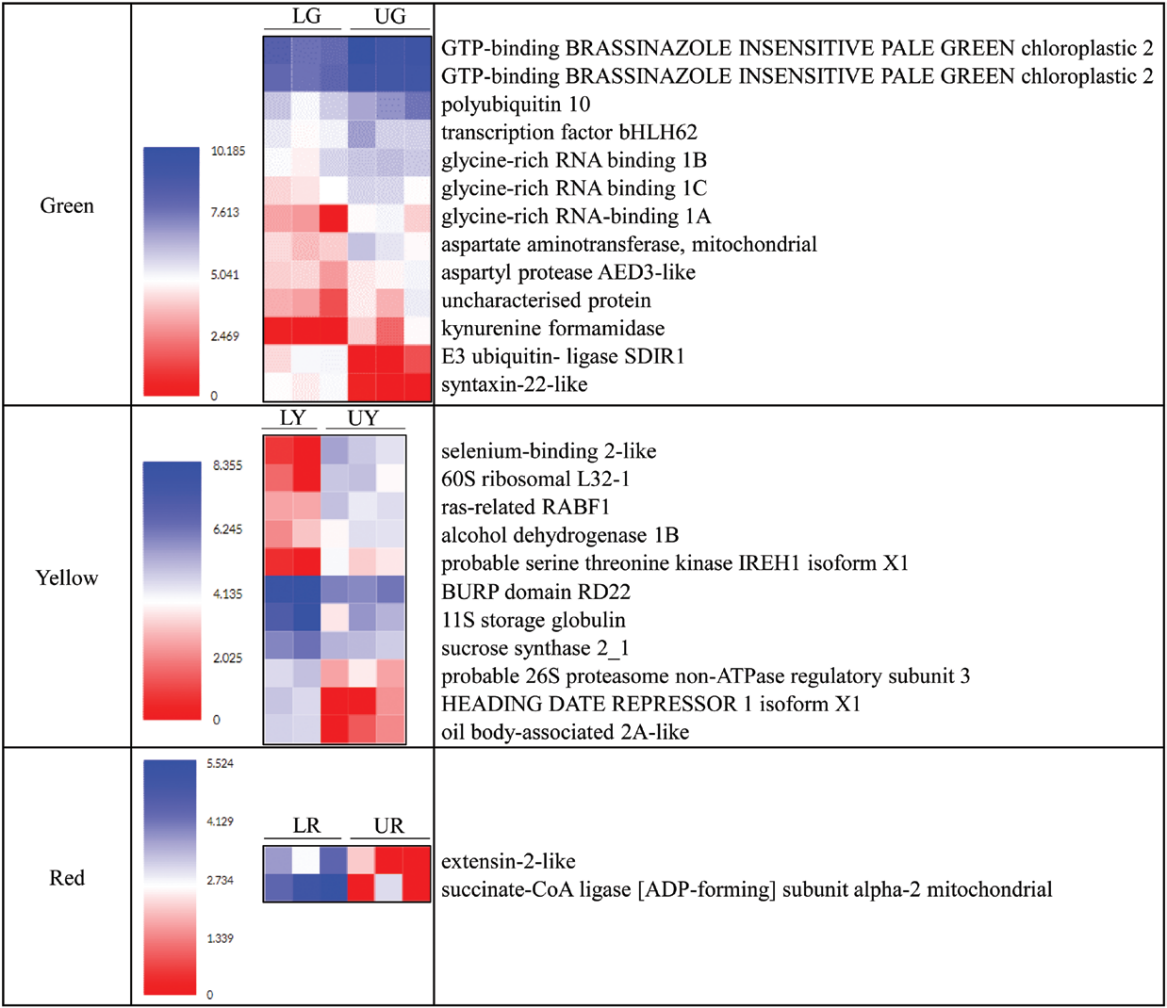
The expression of differentially expressed genes in ripening coffee beans at the two canopy positions with functional annotation. Expression values were transformed with log_2_(TPM+1); L and U, indicate coffee beans from the lower and upper canopy; G, Y, and R indicate coffee beans at the green, yellow, and red stages.

The up- and down-regulated genes defined the rates of change (development) in coffee beans from different canopy positions (Fig. 5). Of the comparisons at the green stage, two transcripts encoding the GTP-binding protein BRASSINAZOLE INSENSITIVE PALE GREEN chloroplastic 2 (BPG2) were expressed ~4-fold more in coffee beans from the upper canopy at the green stage (UG). Greater expression was also shown in transcripts encoding aspartate aminotransferase, mitochondrial (AspAT), polyubiquitin 10 (UBQ10), and glycine-rich RNA-binding 1 (GRP) in UG. Importantly, higher expression was illustrated in transcripts associated with *kynurenine formamidase* (*KFA*) in UG, while this gene was not detected in the lower canopy (LG). In contrast, increased expression in LG was identified in genes linked to *E3 ubiquitin-ligase SDIR1* (*SDIR1*). Genes augmented in expression at the yellow stage in coffee beans from the upper canopy (UY) include *probable serine-threonine kinase IREH1* (*IREH1*). In comparison, up-regulations in LY include genes regulating quality traits, such as *11s storage globulin* (the main protein in the coffee bean), *sucrose synthase 2_2*, and *oil body-associated 2A-like* (regulate oil body size and stores lipids). Importantly, *BURP domain RD22* (*RD22*) was also more highly expressed in LY. At the last stage, only two genes with a known function were identified as up-regulated in coffee beans from the lower canopy (LR), *extensin-2-like* (*EXT2*) and *succinate-CoA ligase [ADP-forming] subunit alpha-2 mitochondrial*.

### Co-expression network

**Fig. 6.**
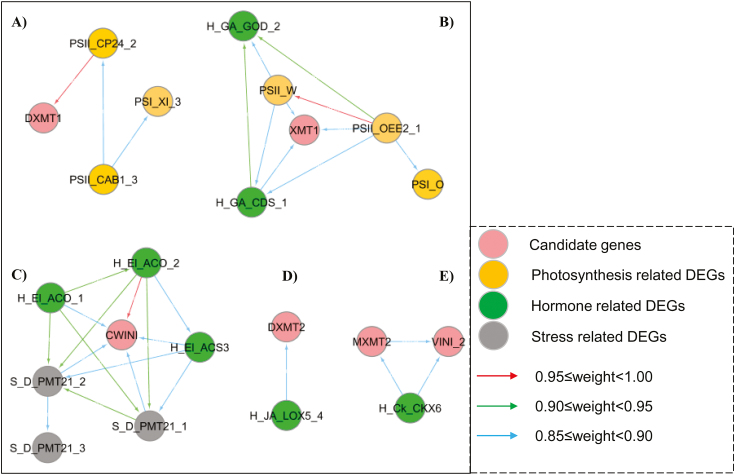
Co-expression network of candidate genes for photosynthesis-, hormone-, and stress-related differentially expressed genes (DEGs). PSI_, PSII_, H_, GA_, JA_, Ck_, El, S_, D_, DEGs related to PSI, PSII, hormone, gibberellin, jasmonic acid, cytokinin, ethylene, stress, and cold response. DXMT1, 3,7-dimethylxanthine *N*-methyltransferase 1; CP24_10A_2, chlorophyll a-b binding protein CP24 10A isoform 2; PSII_CAB1_3, chlorophyll a-b binding protein CAB1 isoform 3; PSI_XI_3, PSI reaction centre subunit XI chloroplastic; XMT1, xanthosine methyltransferase 1; PSII_OEE2_1, oxygen-evolving enhancer protein 2 isoform 1; PSII_W, PSII reaction centre W protein; PSI_O, PSI subunit O; GOD_2, gibberellin 20 oxidase 1-D-like isoform 2; CDS_1, copalyl diphosphate synthase isoform 1; CWINI, cell wall invertase inhibitor; PMT21_1, probable methyltransferase PMT21 isoform 1; PMT21_2, probable methyltransferase PMT21 isoform 2; PMT21_3, probable methyltransferase PMT21 isoform 3; ACO_1, 1-aminocyclopropane-1-carboxylate oxidase isoform 1; ACO_2, 1-aminocyclopropane-1-carboxylate oxidase isoform 2; ACS_3, 1-aminocyclopropane-1-carboxylate synthase 3-like; DXMT2, 3,7-dimethylxanthine *N*-methyltransferase 2; LOX5_4, probable linoleate 9S-lipoxygenase 5 isoform 4; MXMT2, 7-methylxanthine methyltransferase 2; VINI_2, vacuolar invertase inhibitor isoform 2; CKX6, cytokinin dehydrogenase 6-like.

Five networks constructed in this study show the close relationships of candidate quality-associated genes (caffeine and sucrose) with photosynthesis-, hormone-, and stress-related DEGs (Fig. 6). Caffeine genes (*XMT1*, *MXMT2*, *DXMT1*, and *DXMT2*) were co-expressed with photosynthesis-, hormone-, and sucrose-related genes, while sucrose gene expression was related to hormone- and stress-related genes.

*XMT1* was linked to both DEGs linked to *PSII reaction centre W protein* (*PsbW*), *oxygen-evolving enhancer protein 2 isoform 1* (*OEE2_1*), and *ent-copalyl diphosphate synthase isoform 1* (*CPS1_1*). *PsbW* was down-regulated at the yellow stage compared with the green stage in both the upper and lower canopy (Supplementary Dataset S1). The same pattern was observed in *OEE2_1* for beans from the upper and lower canopy. A reduction in the expression of *CPS1_1* was only observed in beans of the upper canopy from the green to the yellow stage. There was no significant difference in *PsbW*, *OEE2_1*, and *CPS1_1* between the two canopy positions. The expression of *DXMT1* was correlated with *chlorophyll a-b binding CP24 10A isoform 2* (*CP24_2*; high confidence: 0.977), while *DXMT2* was related to *probable linoleate 9S-lipoxygenase 5 isoform 4* (*LOX5_4*). The decrease of *LOX5_4* expression was slower in beans from the lower canopy from the green to the yellow stage (1.6-fold) but faster from the yellow to the red stage (2.2-fold). The reverse pattern was identified in beans from the upper canopy, with 2.1-fold and 1.9-fold changes.

In addition, *cytokinin dehydrogenase 6-like* (*CKX6*) was related to both *MXMT2* and the sucrose gene, *VINI_2*, suggesting *CKX6* as a key coordinator between caffeine and sucrose metabolism. A 3.7-fold reduction in *CKX6* expression was observed in LY versus LG, and less change was found in LY versus LG (2.5-fold). Compared with the other candidate genes, *CWINI*, involved in sucrose accumulation, cooperated with more DEGs. This includes 1-*aminocyclopropane-1-carboxylate oxidase isoform 1* (*ACO_1*) and *2* (*ACO_2*; high confidence: 0.95) and *1-aminocyclopropane-1-carboxylate synthase 3-like* (*ACS3*) as well as *probable methyltransferase PMT21 isoform 1* and *2* (*PMT21_1* and *PMT21_2*). All these co-expressed genes showed increased expression in the upper canopy from the green to the yellow stage, compared with the lower canopy.

## Discussion

### Improved quality and candidate genes selected

Increased aroma intensity explains improved organoleptic coffee quality (Bhumiratana *et al.*, 2011; Muschler, 2001). From the chemical analysis, an increased sucrose, trigonelline, and caffeine content contributes to this aroma (organoleptic) improvement in coffee beans from the lower canopy. Unfortunately, the stages studied here did not explain the increased level of caffeine accumulation in coffee beans from the lower canopy. This means that the variation in caffeine gene expression may not be captured by the developmental stages studied here. However, longer maturity offers more trigonelline and sucrose accumulation in coffee beans from the lower canopy contributed by candidate genes regulating their metabolic pathways. The crop management of this study is similar to the shade management in previous studies (Geromel *et al.*, 2006; 0–80% shade levels on the same K7 plant in Odeny *et al.*, 2014; 45% shade in Vaast *et al.*, 2006 and Worku *et al.*, 2018). The upper canopy beans have more sunlight exposure, while the lower have less (Supplementary Fig. S1). The difference in crop management is that the sample in this study was collected from the same plant, which has fewer biomass variations (due to factors such as soil, climate, or other differences). The same trend of increased caffeine and trigonelline was found in coffee grown in shade levels at 50–80%; however, sucrose content has a reverse trend (Odeny *et al.*, 2014). As the upper canopy coffee beans grow faster, they become source tissues earlier and their sucrose may contribute to other sink tissues, while the lower canopy beans had a longer maturity to accumulate sucrose as sink tissues, either by loading from the source tissues of the same plant or by further sucrose resynthesis (suggested by higher *SPS* expression).

Many of the candidate genes exhibited expression consistent with that reported in the previous RT-PCR studies in both Arabica and Robusta coffee, for example the dominant caffeine genes, *XMT1* and *DXMT2*. Consistency was also observed in genes related to sucrose metabolism in Arabica coffees (genotype CCCA02 or CCCA12) (Privat *et al.*, 2008), such as *SUS* and *CWINI* (Koshiro *et al.*, 2006; Mizuno *et al.*, 2003; Perrois *et al.*, 2015): (i) the expression pattern of *SUS2*, *SPS1*, *SPS2*, *SPP1*, and *VIN* was the same for both Arabica genotypes [note: *SUS1* from Arabica coffee (accession number AM087674.1# was the subgenome copy of *SUS2* from Robusta coffee (DQ834312.1); (ii) a uniform expression with peak expression at the intermediate stage was observed for *CIN* in both the Arabica and Robusta genotypes. The consistent expression of these genes validates the RNA-Seq analysis in this study and suggests value in modifying these genes to control sucrose accumulation in Arabica and/or Robusta coffee.

The main genes regulating improved sucrose concentration through the metabolic pathway include *CIN*, *SPS2_2*, *SPS1*, *SUS2_2*, *VINI_2*, and *VIN_2*. Sucrose is the carbon backbone used to form the major components in the plant; together with fructose, UDP-glucose (produced from sucrose degradation) can be used for the formation of cell wall polysaccharides, proteins, and lipids (Ruan, 2014). Cell wall invertase inhibitor (CWINI) has been reported to suppress CWIN activity (Ruan, 2014), and inhibition of *CWINI* enhanced tomato CWIN activity (2-fold) and seed weight (Jin *et al.*, 2009). From our previous studies, we understand genes associated with many essential components mainly accumulate from the green stage, for example galactomannan (~50% of cell wall polysaccharides), 11S (~45% of protein), and triacylglycerol (78% lipids) (De Castro and Marraccini, 2006; Cheng *et al.*, 2018) (Figs 2, 3; Supplementary Figs S2, S3). Corresponding to this trend, higher activity was identified in the expression of sucrose degradation genes, *SUS1* and *CWIN* (suggested by *CWINI*), which break down sucrose (to UDP-glucose and fructose) to form galactomannan, lipids, and protein, major components in the bean. CIN mediates sugar homeostasis under low SUS activity and it alternatively degrades sucrose in the cytoplasm (to glucose and fructose) (Ruan, 2014). Therefore, once the expression of *SUS1* decreased significantly, that of *CIN* and *SUS2* (much lower level compared with *SUS1*) took over the role of sucrose degradation for further formation of downstream components, such as CGAs or other non-dominant metabolites.

More specifically, higher expression of *C4H4* and *C3'H* in this study suggested that 5-CQA and di3,5-CQA had a higher level of further or delayed accumulation in lower canopy beans at the green stage. The main accumulation of 5-caffeoyl quinic acid (5CQA; making up the majority of CGAs) was before the green stage (~180 DAF) and it was isomerized to minor isomers, such as 4-CQA. However, cool temperature delays peak 5-CQA formation, while it has a weak negative correlation with solar irradiation; however, isomer accumulation is negatively correlated with temperature (Joët *et al.*, 2010*b*). Since the difference in temperature from the upper to the lower canopy decreased from 1 °C to 4 °C (Wintgens, 2004), a parallel improvement was likely to improve feruloyl quinic acid (FQA) content instead of minor isomers in the lower canopy beans, suggested by increased expression of *CCoAOMT1*, *CCoAOMT5*, and *HCTa*.

### Improved quality with less sunlight and slower growth

According to the analysis of DEGs, less sunlight exposure produced a slower development in coffee beans from the lower canopy. DEGs related to photosynthesis, hormone, and stress provided more details to understand this mechanism. At early development (especially at the green stage), UG had a higher level of photosynthesis activity and a possibly lower degree of maturity, as suggested by improved *BPG2* expression (5-fold). *BPG2* was reported to control light regulation of chloroplast photosynthetic protein translation and chlorophyll formation (Komatsu *et al.*, 2010; Kim *et al.*, 2012; Nakano *et al.*, 2013). Increased expression of *BPG2* in UG is likely to result from a higher level of sunlight exposure in the upper canopy. When green tissues in coffee beans turned different colours at later development (yellow stage), the activity of photosynthesis reduces. However, beans from the lower canopy had a lower photosynthetic loss, as indicated by enhanced expression of *RD22*. *RD22* was reported to be expressed at the early to mid-stage of seed development and is up-regulated in response to dehydration, abscisic acid (ABA), and salt stress, and suppresses chlorophyll degradation (Yamaguchi-Shinozaki and Shinozaki, 1993; Ding *et al.*, 2009; Harshavardhan *et al.*, 2014). When coffee beans turn yellow, there is a transition from the milky stage (in mucilage) to the hardening stage in the beans due to moisture loss (Cheng *et al.*, 2019). The almost five times elevated *RD22* expression in LY compared with UY illustrated that a stronger dehydration resistance and less chlorophyll degradation was present in LY that facilitated continued growth until the red stage. Moreover, LG used strategies to approach a higher development speed under an environment with less sunlight, while this did not seem necessary for UG. This is suggested by KFA expression, which was not detectable in LG but was more highly expressed in UG. KFA is involved with the tryptophan catabolic process to kynurenine, which can inhibit a tryptophan-dependent pathway that is necessary for auxin biosynthesis in plant shade avoidance (Tao *et al.*, 2008; He *et al.*, 2011; Ljung, 2013).

More photosynthesis boosts growth in UG, supported by higher expression of genes linked to the plant hormones, such as *UBQ10* and *GRP* isoforms [related to salicylic acid (SA) accumulation]. Up-regulation of *UBQ10* was determined in Arabidopsis in response to SA (Blanco *et al.*, 2005). GRP is more abundant in young tissues; it suppresses cell death, is enhanced by SA, and responds to stress, such as dehydration, cold, and salt (Condit and Meagher, 1987; Sachetto-Martins *et al.*, 2000; Zchut *et al.*, 2003; Lu and Chen, 2005). Rapid growth in UG was also suggested by greater expression of *AspAT* and the transcription factor gene *bHLH62* in UG and of *IREH1* in UY. This is because *AspAT* is light dependent and feeds the amino acid pool for nitrogen assimilation in terms of carbon and energy metabolism (Schultz and Coruzzi, 1995; Nomura *et al.*, 2005; Lee *et al.*, 2008), and *bHLH62* plays a role in a broad range of plant growth, stress (heat), and hormone [signalling of jasmonic acid (JA)] responses and metabolite synthesis (Kazan and Manners, 2013; Spadoni *et al.*, 2015; Zhang *et al.*, 2016). In addition, *IREH1* was one of the key genes regulating growth (Oyama *et al.*, 2002). On the contrary, growth in coffee beans from the lower canopy was inhibited at an early stage by *SDIR1*, which was proved to be a positive ABA regulator of signalling, and overexpression of *SDIR1* leads to enhanced ABA (growth inhibition) (Zhang *et al.*, 2007; Xia *et al.*, 2012; Zhang *et al.*, 2015). This provided evidence that extra ABA was probably produced to suppress bean growth in the lower canopy. However, longer maturity was shown by increased expression of EXT2 in coffee beans from the lower canopy at later development (LR) associated with cell wall strengthening or embryo maturity. This is because the development of coffee seed starts from the perisperm to endosperm and then the embryo (De Castro and Marraccini, 2006). Moreover, extensin plays a key role in plant growth and development by strengthening the cell wall and supporting the normal development of the embryo by modifying the cell plate in cytokinesis (Guo *et al.*, 2014).

### Interaction of sunlight, growth, and chemical accumulation

Importantly, the gene co-expression illustrated that genes related to caffeine (*MXMT2*) and sucrose metabolism (*VINI_2*) were correlated and interact with *CKX6*. CKX inactivates cytokinin, and delayed or reduced *CKX* leads to higher growth (Werner *et al.*, 2003). A more rapid decrease of *CKX6* expression from LG to LY suggested further development of coffee beans from the lower canopy. Light is required in caffeine synthesis, and the optimal requirement is very low (Kumar *et al.*, 2015). From this study, this phenomenon is explained by the co-expression of caffeine genes with photosynthesis genes (*PsbW*, *OEE2_1*, and *CP24_2*). The expression of *PsbW* is light mediated (Lorković *et al.*, 1995), and *OEE2_1* encodes PsbP and controls PSII and response to biotic stress (Lorković *et al.*, 1995; Seidler, 1996; Förster *et al.*, 2006). CP24 functions in the light harvesting of PSI; plants with no CP24 have reduced electron transport and limited growth (De Bianchi *et al.*, 2008). Moreover, caffeine genes are likely to be developmentally regulated by CPS1_1 and LOX5_4. CPS regulates gibberellin biosynthesis, while LOX has a diverse function in plant development, fruit ripening, and as a seed storage protein; it processes hydroperoxide fatty acids to form JA (which inhibits growth) (Kolomiets *et al.*, 2000; Guo *et al.*, 2015; Rameshwari *et al.*, 2015). A different *LOX5* accumulation pattern in bean ripening indicates its role in seed maturity regulation in the two canopy positions. According to previous studies, *CWINI* also negatively mediates cytokinin by a method other than inhibition of CWIN in tobacco leaf (Lara *et al.*, 2004). Results from this study provided further details through the co-expression network; *CWINI* co-expressed with more DEGs highlighted its important role in bean growth and stress response. These genes are involved in ethylene formation, *ACO_1*, *ACO_2*, and *1-ACS3* (White *et al.*, 1994; MCcarthy *et al.*, 2001), as well as the dehydration response (*PMT21_1* and *PMT21_2*) (Kiyosue *et al.*, 1994). Increased expression of these genes from UG to UY would result in more ethylene, accelerated maturity, and a greater dehydration response.

### Conclusion

Climate change threatens many world high-quality coffee plantations. Genetic adaptation of coffee production in a wider range of environments is required to ensure a sustainable supply of high-quality coffee. This study reveals that the lower canopy produced high-quality coffee beans with more intense coffee aroma (improvement in organoleptic coffee quality) associated with improved sucrose, trigonelline, and caffeine accumulation in the bean. Increased chemical components in these beans were linked to a longer period of accumulation and increased expression of candidate genes in the trigonelline (*CTgS2*) and sucrose (*CIN*, *SPS2_2*, *SPS1*, *SUS2_2*, *VINI_2*, and *VIN_2*) metabolism pathways. Starting from a higher maturity stage, beans in the lower canopy had a longer maturity, possibly controlled by growth and photosynthesis genes at the green stage to delay growth, reduce photosynthesis loss at the yellow stage, and extend growth at the red stage. However, beans from the upper canopy grew rapidly from a lower maturity level at the green stage, with elevated chlorophyll formation as well as carbon and energy consumption. Ultimately, we have identified gene markers for bean quality improvement. These include candidate genes from trigonelline (*CTgS2*) and sucrose (*CIN*, *SPS2_2*, *SPS1*, *SUS2_2*, *VINI_2*, and *VIN_2*) metabolism pathways, DEGs (such as *SDIR1*, *RD22*, *BPG2*, *AspAT*, *GRPs*, *bHLH62*, and *UBQ10*), and co-expressed genes (including *CKX6*) related to quality differentiation in the two canopy positions that could be manipulated in future breeding to produce high-quality coffee beans. Moreover, an adaptation of coffee production to new environments may be facilitated by selection of genotypes with appropriate levels of expression of these genes contributing to coffee quality under the target environmental conditions.

## Data availability

The transcriptome data set can be accessed from the EMBL with accession numbers PRJEB24137 and PRJEB24850.

## Supplementary data

Supplementary data are available at *JXB* online.

Table S1. Sensory lexicon for coffee and the definitions and sensory reference standards.

Fig. S1. Experimental design.

Fig. S2. Expression of key genes in the main biosynthetic pathway of cell wall polysaccharides, adopted from (Cheng *et al.*, 2019).

Fig. S3. Expression of key genes regulating the formation of lipids and protein.

Fig. S4. PCA analysis of gene expression in three ripening stages of coffee beans from the upper (U) and lower canopy (L).

Dataset S1. Differentially expressed genes associated with photosynthesis, hormones, and stress.

eraa151_suppl_supplementary_dataset_S1

eraa151_suppl_supplementary_table_S1_figures_S1-S4

## Abbreviations

ABA: abscisic acid
ACO: 1-aminocyclopropane-1-carboxylate oxidase
ACS3: 1-aminocyclopropane-1-carboxylate synthase 3-like
AspAT: aspartate aminotransferase, mitochondrial
bHLH62: basic helix–loop-helix protein 62
BPG2: BRASSINAZOLE INSENSITIVE PALE GREEN chloroplastic 2
CAB: chlorophyll a-b binding protein CAB1
CCoAOMT: caffeoyl-CoA 3-*O*-methyltransferase
CGA: chlorogenic acid
C4H: trans-cinnamate 4-hydroxylase
C3'H: *p*-coumaroyl CoA 3-hydroxylase
CIN: cytosolic invertase
CKX6: cytokinin dehydrogenase 6-like
CP24_2: chlorophyll a-b binding CP24 10A isoform 2
CPS1_1: ent-copalyl diphosphate synthase isoform 1
CQA: caffeoyl quinic acid
CTgS2: trigonelline synthase 2
CWIN: cell wall invertase
CWINI: cell wall invertase inhibitor
DAF: days after flowering
DEG: differentially expressed gene
DXMT: 3,7-dimethylxanthine *N*-methyltransferase
EXT2: extensin-2-like
FQA: feruloyl quinic acid
GRP: glycine-rich RNA-binding
HCT: hydroxycinnamoyl-CoA:shikimate/quinate hydroxycinnamoyl transferase
HQT: hydroxycinnamoyl-CoA quinate hydroxycinnamoyl
IREH1: probable serine-threonine kinase IREH1
JA: jasmonic acid
KFA: kynurenine formamidase
LOX5_4: probable linoleate 9S-lipoxygenase 5 isoform 4
MXMT: 7-methylxanthine methyltransferase
OEE2_1: , oxygen-evolving enhancer protein 2 isoform 1
PAL: phenylalanine ammonia lyase
PCA: principal component analysis
PsbW: PSII reaction centre W protein
PMT21: probable methyltransferase PMT21
PSI_O: PSI subunit O
PSI_XI: PSI reaction center subunit XI chloroplastic
RD22: BURP domain RD22
RNA-Seq: RNA sequencing
RT-PCR: real-time PCR
SA: salicylic acid
SDIR1: E3 ubiquitin- ligase SDIR1
SPP: sucrose phosphate phosphatase
SPS: sucrose phosphate synthase
SUS: sucrose synthase
TPM: transcripts per kilobase million
UBQ10: polyubiquitin 10
VIN: vacuolar invertase
XMT: 7-methylxanthosine synthase
