# Supplementary material for: Slower development of lower canopy beans produces better coffee
Source: J Exp Bot. 2020 Mar 24;71(14):4201–14. doi: 10.1093/jxb/eraa151 (PMC7337091; doi:10.1093/jxb/eraa151)
Supplement: eraa151_suppl_supplementary_table_S1_figures_S1-S4 [file eraa151_suppl_supplementary_table_s1_figures_s1-s4.pdf]

Slower development of lower canopy beans produces better coffee

Bing Cheng*[1,2], Heather E. Smyth[1], Agnelo Furtado[1], Robert J. Henry*[1]

[1]Queensland Alliance for Agriculture and Food Innovation, The University of Queensland, St Lucia, QLD 4067, Australia

[2] Current address: Crop Production and Biostimulation Laboratory, Universite libre de Bruxelles, Campus Plaine CP242, Bd du Triomphe, 1050 Brussels, Belgium

*Corresponding to chengbing0404@hotmail.com; robert.henry@uq.edu.au

Supporting Table S1 Sensory lexicon for coffee and the definitions and sensory reference standards.

	Sensory attributes	Definition	Reference standards
Aroma	Overall aroma intensity	The overall aroma intensity of the sample.	NA
	Citrus zesty	A sharp lifted citrus, zesty aroma detected when first lifting the lid.	Citrus zest
	Flowery	A heady floral, fragrant note.	Jasmine tea
	Blackcurrant / berry	A fruity, blackcurrant or red currant aroma, berry-like.	Blackcurrant jam
	Herby / grassy	A slightly dried herbaceous note, herby and grassy almost like pipe tobacco.	Green snow pea
	Chocolate	A chocolate note, cocoa-like.	Dark chocolate grated
	Burnt	An acrid, burnt and charry note.	Ash from the smoker
	Smoky	A smoky note with a little ashy.	Ash from the smoker
	Toasted	A lighter toast aroma, pleasant like freshly toasted bread.	Toasted white bread
	Cereal / caramel	A sweet caramel aroma like cereal and molasses, dark brown sugar.	Original caramel, oat bran, pinch of dark brown sugar, drops of water
	Roasted nutty	A roasted nutty aroma like roast peanuts or pecans	Crushed hazelnuts (with skins) and roasted peanuts.
	Woody	Aroma of wood chips, cedar and fragrant almost medicinal and clove-like	Essential oil essence in wood chips
	Pepper spice	A gragrant spicy pepper aroma, like black pepper, Sichuan pepper, star anise.	Black pepper and other spices crushed
	Aroma attributes	Definition	Reference standards
	‘other’ aroma		NA
Flavour/mouth feel	Tart / sourness	The sourness of the sample	Nil
	Saltiness	The saltiness of the sample	Nil
	Bitterness	The bitterness of the sample	Nil
	Astringency	The astringency, drying sensation in the mouth and cheek pouches	Nil
	Oiliness	A mouth coating oiliness sensation	Nil
	Overall flavour intensity	The overall intensity of flavour sensations	Nil
	‘other’ flavour		NA

Experimental design

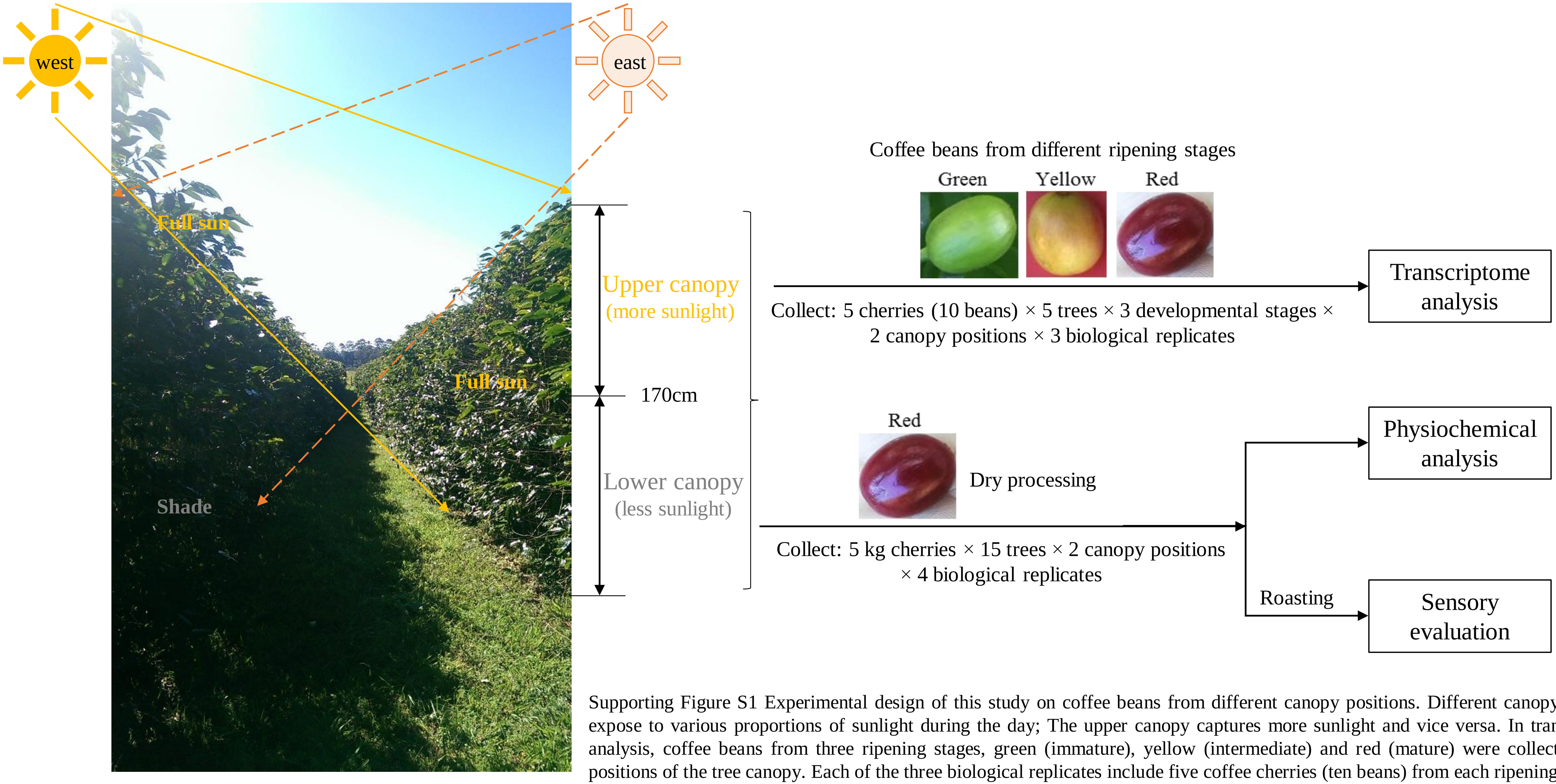

Supporting Figure S1 Experimental design of this study on coffee beans from different canopy positions. Different canopy positions expose to various proportions of sunlight during the day; The upper canopy captures more sunlight and vice versa. In transcriptome analysis, coffee beans from three ripening stages, green (immature), yellow (intermediate) and red (mature) were collected at two positions of the tree canopy. Each of the three biological replicates include five coffee cherries (ten beans) from each ripening stage and canopy position of individual trees (five trees altogether). In the phenotypic analysis, red beans were collected for dry processing and then physiochemical analysis. Roasting were conducted before sensory evaluation. Each coffee sample of the four biological replicates from individual canopy position has 5kg cherries collected from 15 randomly selected trees of the same row.

Supplementary Figure S2 Expression of key genes in the main biosynthetic pathway of cell wall polysaccharides, adopted from Cheng et al. 2020. LG, LY, LR indicate coffee beans at green, yellow and red stages from the lower canopy; UG, UY, UR mean coffee beans at green, yellow and red stages from the upper canopy. CESA, cellulose synthase; UGE, UDP-glucose 4′-epimerase; GMGT, galactomannan galactosyltransferase; α-Gal, alpha-galactosidase; UGDH, UDP-Glucose dehydrogenase; UGA4E, UDP-glucuronic acid 4′-epimerase; GAUT, galacturonosyltransferase; MS, mannan synthase; MPGT, mannose-1-phosphate guanylyltransferase.

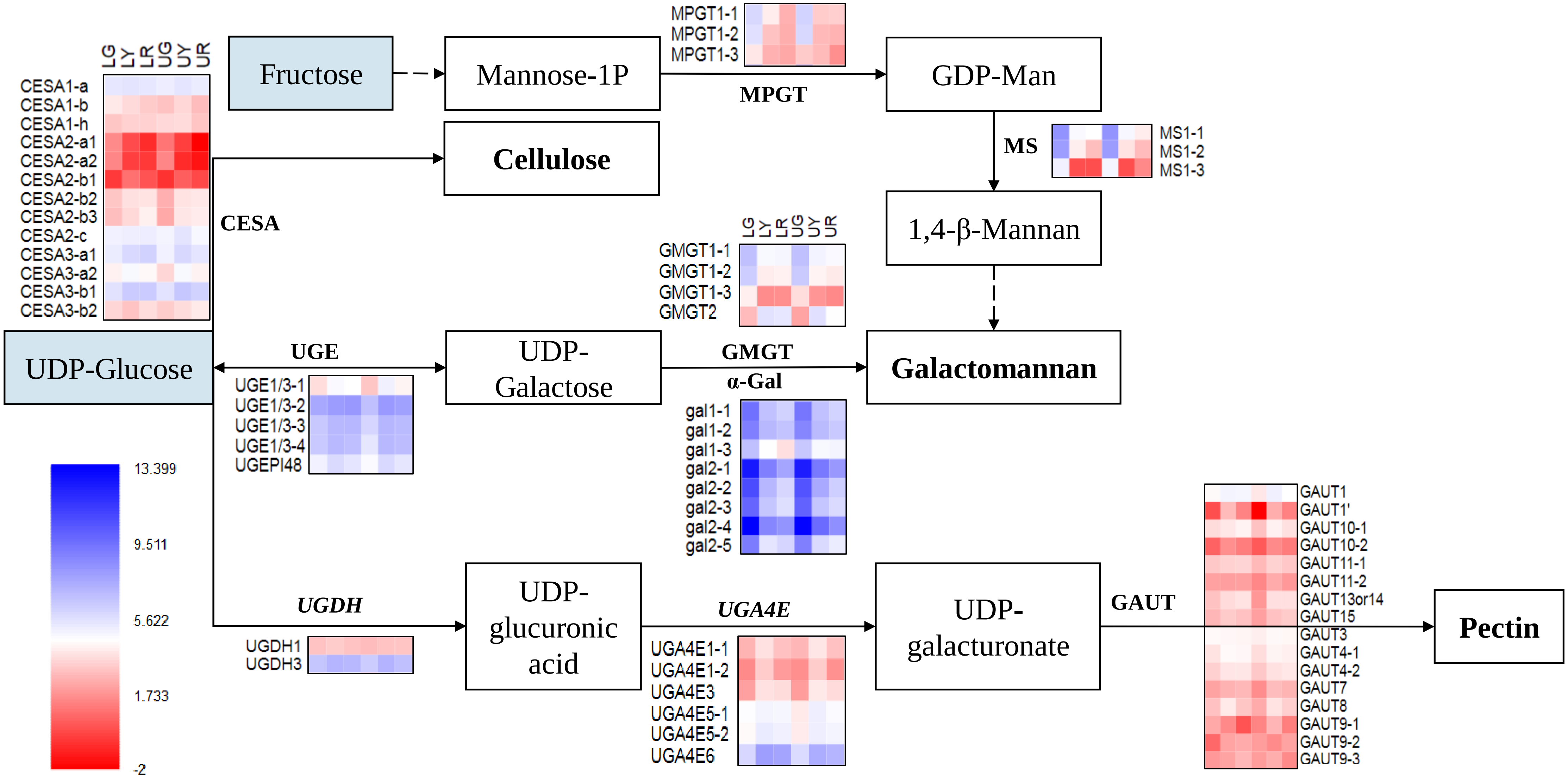

Supplementary Figure S3 Expression of key genes regulating the formation of lipids and protein. Hotmap is based on log2 transformed expression value. LG, LY, LR indicate coffee beans at green, yellow and red stages from the lower canopy; UG, UY, UR mean coffee beans at green, yellow and red stages from the upper canopy. TAG, triacylglycerol; OLE, oleosin (TAG synthase); 11S, 11S globulin; -1,-2,-3 means different transcript isoforms.

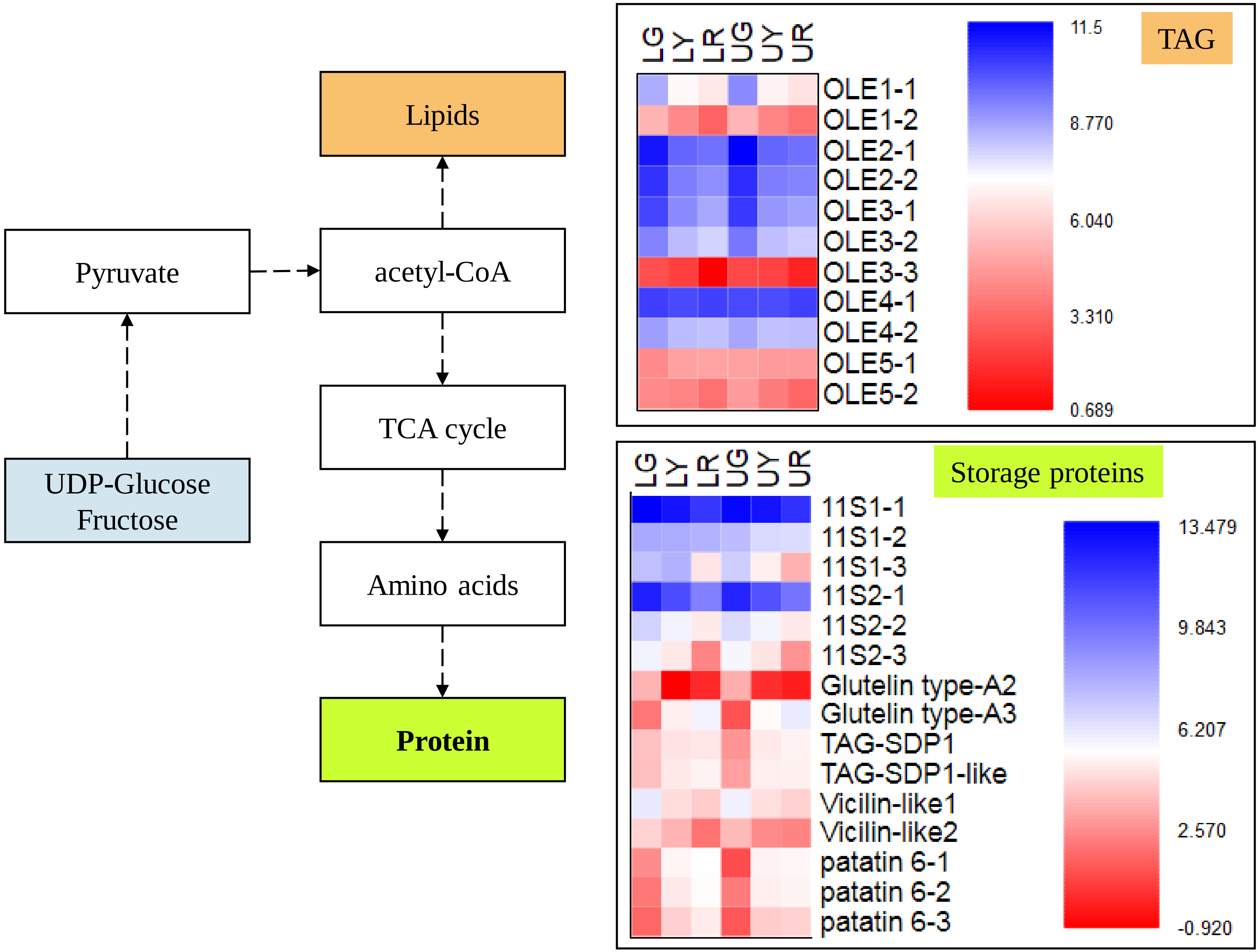

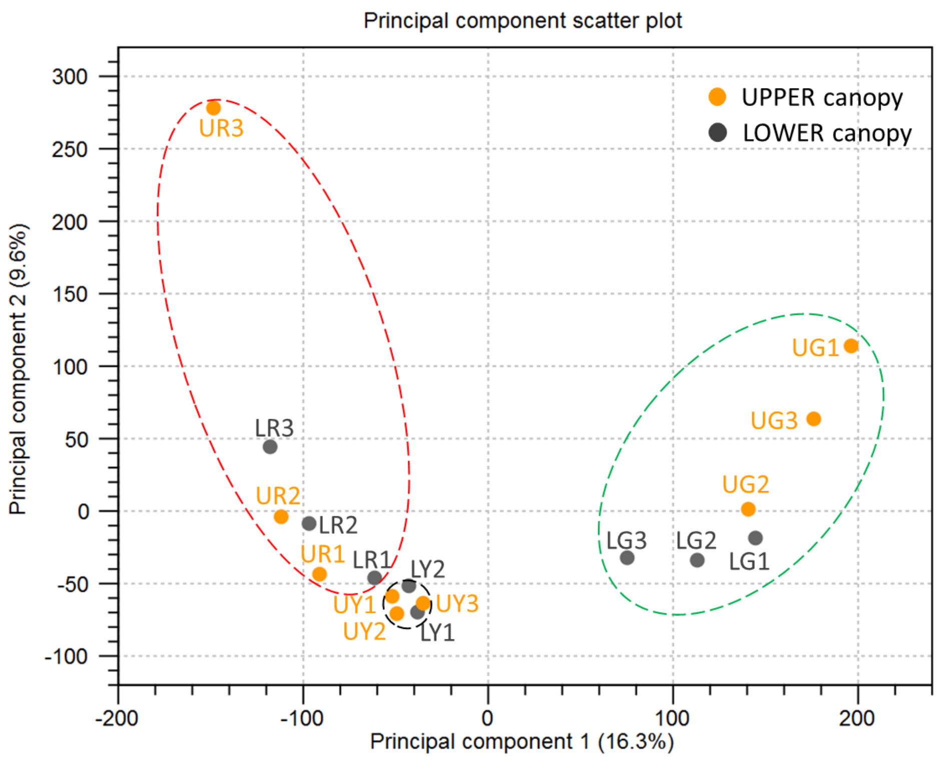

Supplementary Figure S4 PCA analysis of gene expression in three ripening stages of coffee beans from the upper (U) and lower canopy (L). G, Y, R, green, yellow and red stages depends on the pericarp colour.
